# Gene polymorphism among hypertensive patients with coronary artery disease

**DOI:** 10.6026/97320630018239

**Published:** 2022-03-31

**Authors:** D Selvakumar, C.K Vijayasamundeeswari, E Gnanadesigan, N Sivasubramanian

**Affiliations:** 1 Department of Biochemistry, Vinayaka Mission's Kirupananda Variyar Medical college and Hospitals, Salem, Vinayaka Mission's Research Foundation (Deemed to be University), Salem -636 308, Tamilnadu, India; 2 Department of Physiology, Nootan Medical College & Research Centre, Sankalchand Patel University, Visnagar - 384315, Gujarat, India; 3 Department of Psychiatric Nursing, Nootan College of Nursing, Sankalchand Patel University, Visnagar- 384315, Gujarat, India

**Keywords:** IL-6 -174, IL-6-572, CAD, hypertension, gene polymorphism

## Abstract

The obstruction of the coronary arteries causes Coronary Artery Disease (CAD). It has been reported that interleukin-6 gene is related to the development of cardiovascular diseases such as atherosclerosis and coronary artery disease. This was due to the large variability and short half-life of interleukin 6 (IL-6). There are few studies on the link between interleukin 6 and CAD on the patients with hypertension. Therefore, goal of this study was to see if there is a link between IL-6 gene polymorphisms and coronary artery disease with hypertension patients. The polymorphisms were carried out by polymerase chain reaction–restriction fragment length polymorphism (PCR-RFLP). The data was determined for statistical significance using chi-square analysis. A significant difference was found in the GG genotype of IL-6 -174, which was more frequent in cases of CAD (48.67 %) than in controls (8%) and 95% CI was 0.473455 - 0.500326; P<0.010620511. The GG genotype of IL-6-572C/G polymorphism was more frequent in cases of CAD (42.6%) compared with controls (8%) and 95% CI 0.386724 - 0.480945; P<0.017939631). likewise, significant association of variant allele G with CAD patients was reported. Hypertension was significantly higher among patients as compared to controls (P<0.022847535). Our findings indicated that both gene polymorphisms may be associated with development of CAD.

## Background:

Coronary artery disease (CAD) is the leading cause of morbidity and mortality in the world, and are associated with diabetes, dyslipidemia and hypertension, and it is common among smoking and alcohol consumption population. CAD involves disorders of the cardiovascular and blood circulation systems. Such disease is also an enormous consumption of medical resources, particularly in a promptly aging society. Although the etiology of coronary heart disease seems to be complicated by multifactorial, the uttermost important pathogenic factor is coronary atherosclerosis. Atherosclerosis is a chronic inflammatory reaction related with various pathophysiological reactions inside the vascular wall. Inflammation is the main factor in the progression of atherosclerosis and is reported to be the main risk factor; atherosclerosis is orchestrated by a range of cytokines, which regulate all stages of the disease. Among the cytokines, inflammatory cytokines are important and related with atherosclerotic risk in a different clinical experience [1]. The inflammatory cytokines of IL6, IL31 and TNF-a are associated with CHD and gene polymorphism was occurred in these cytokines [2]. Many polymorphisms of the IL-6 promoter have been studied in healthy and unhealthy people. Two SNPs, the common –IL-6-174 G/C variant and the less common –IL-6-572C/Gallele have been reported in detail. The previous study shows that –IL-6-174G/C and -572C/G polymorphisms in the IL-6 gene may increase plasma concentrations of IL-6. It was also studied that the polymorphisms can affect the transcription and plasma levels of IL-6 [3,4]. However, Bennermo et al.(2004)[5] were reported that the -IL-6-174 G/C polymorphism was not involved with cardiovascular diseases, same time -IL-6-572 C/G polymorphism was associated with a CHD. In this situation a more concise report can be obtained by summarizing the research results systematically and accurately, as it is necessary in order to prevent the risks related to CAD and the high mortality rate of the disease. The purpose of this study is to assess the relationship between IL-6 gene polymorphism and CAD. We analyzed the presence of IL-6 genotypes and alleles among 150 CAD patients and 25 control subjects.

## Methods:

### Study population:

A case-control study was designed. The case group consisted of 150 patients fulfilling the CAD diagnostic criteria of ACCC/ AHA guidelines. These criteria were: two angina episodes at rest or an angina episode lasting more than 20 minutes in the last 48 hours, electrocardiographic (ECG) ST-segment elevation during angina. Using the result of ECG, Those who had obstructive lesions of more than 50%in one or more coronary arteries were considered to have CAD. Arterial blood pressure was recorded in sitting posture with a random zero mercury sphygmomanometer, after the subject had been seated for 5 minutes. The control group comprised 25 individuals without history of cardiovascular disease, who attended the hospital for reasons unrelated to CAD. The case individuals were selected in the cardiology unit of PES medical college super specialty hospital, Kuppam, Andhra Pradesh. The present study was approved by institutional Ethics committee (Approval No: PESIMSR/IHEC/52/2017-18), and an informed consent was taken from all the subjects after explaining the test procedures and goal of the study in local language.

### Determination of IL-6 gene polymorphism:

Venous blood samples were collected in EDTA tubes to obtain genomic DNA. This was isolated from leukocytes using an Aura biotech Genomic DNA Purification Kit. The DNA region containing the polymorphic site was amplified by polymerase chain reaction (PCR). A final volume of 25 µl: 12.5 µL of Master Mix (Smart prime), 0.5 µL of FW primer and 0.5 µL of RV primer (Eurofin), 3 µL of DNA and 8,5 µL of nuclease-free water was obtained for each sample (G-174C and G-572C polymorphisms). Cycling parameters for Il-1 were 3 minutes at 94°C and then 35 cycles at 95°C for 2 minutes, at 53°C for 1 minute and at 74°C for 1 minute, followed by a final extension at 72°C for 5 minutes. Cycling parameters for both polymorphisms, 10 minutes at 94°C and then 35 cycles at 95°C for 1 minute, at 55°C for 1 minute 35 seconds and at 72°C for 1 minute, followed by a final extension at 72°C for 10 minutes [6]. The amplification products were digested with specific restriction enzymes and then separated by horizontal electrophoresis in agarose gels (2.5% w/v) with EtBr stained gel. Primers and enzymes used in the study are detailed in Table 1(see PDF).

### Statistical analysis:

A chi-square test was used to analyze whether the study population was at Hardy-Weinberg equilibrium for the polymorphisms studied.

## Results:

The case-control population consisted of subjects with average age between 30 and 71 years above. Presently, two polymorphic studies were carryout in CAD patients by using PCR techniques. In case of IL-174G/C studies, GG (Mutant homozygous) genotype was frequently observed in patient's samples than control samples, and followed by CG genotype (Heterozygous), which was observed from 49 patients. The lowest prevalence of genotype was CC, which were in 28 patients. Male gender was predominant in both cases and controls. A significant difference was found in the GG genotype of IL-6 -174, which was more frequent in cases of CAD (48.67 %) than in controls (8%) and 95% CI was 0.473455 - 0.500326; P<0.010620511. Furthermore, significant value also was observed in GG genotype in hypertension patients, which was more frequent in hypertension patients, (46.42 %) and 95% CI was 0.415222 - 0.511738; P<0.011574616 (Table 2-4, Figure 1-3 - see PDF). The GG genotype of IL-6-572C/G polymorphism was more frequent in cases of CAD (42.6%) compared with controls (8%) and 95% CI 0.386724 - 0.480945; P<0.017939631). Moreover, GG genotype was frequently observed in hypertension patients than non hypertension patients and also showed with significant value, the 95% CI was 0.461162 - 0.642661; P<0.022847535. In present study, most of the mutant homozygous is observed in hypertension patients than non hypertension patients (Table 5 - 7, Figure 1-3 - see PDF).

## Discussion:

Cardiovascular diseases, including coronary artery disease, have very complex causes. However, the influence of environmental and genetic factors on the occurrence of these diseases has been confirmed. The risk of coronary artery disease is moderately increased due to genetic factors. However, the genetic pattern that greatly increases the risk of CAD remains unknown [7]. In spite of the genetic susceptibility of exposing individuals to abnormal levels of atherosclerosis also increases the risk of CHD [8]. The advancement of biomolecular technology has made it possible to perform numerous polymorphism research studies. The polymorphism of cytokine genes, including interleukin-6, has revealed that the amount of these proteins produced varies from person to person. Different interleukin-6 (IL-6) protein expression pathways have also been identified to play specific biological functions in the development of CHD damage [9]. CHD associated myocardial dysfunction have also been reported with increased levels of various pro-inflammatory cytokines including IL-1α, IL-1β, IL-6, IL-31, TNF-α and INF-γ [10-11]. In earlier study, IL-6 174 G/C polymorphism and CHD were inconsistent. Some studies, such as Bennet [12, 13] did not find the association between the polymorphism and risk of CHD. However, Zheng [14] were determined that 572C allele was associated with a decreased risk of CHD when compared with the 2572G allele, same time the 2174C allele was not significantly associated with CHD risk. The maximum of these studies had been accomplished in western nations on western populations. Further, unique traits among studies, which include ethnicity, ailment diagnosis standards, sources of controls, sample size, can introduce heterogeneity, making it hard to interpret the consequences of association studies. It would accordingly be hard to locate the study correlation among the polymorphisms of the IL-6 gene and CHD.

A meta-analysis was conducted to assess a large number of events in order to determine the source of heterogeneity and to provide a better picture of the findings of all prior research. Many research scholars were interested in the links between the IL-6-572 C/G and IL-6-174 G/C polymorphisms and hypertension, but they couldn't come to conclusion regarding its association with coronary artery disease. To the best of our knowledge, this is the first analysis conducted to determine the association between the IL-6-572 C/G IL-6-174 G/C and polymorphism and hypertension in our hospital. We found that the G allele was more common in patients than in healthy controls, implying that the both polymorphism could be a risk factor for hypertension induction. This was supported the previous researches, which was associated between the development of hypertension and the -572 C/G rather than -174 G/C polymorphism. This study has some potential limitations because we did not analyze the plasma levels of IL-6. Hence, a single measurement might be not predictive of disease in a given individual. For this reason, our study was focused on the analysis of a polymorphism that genetically determines the plasma levels of IL-6 only. Furthermore, both polymorphisms were observed from control patients also, but more predominant in hypertension patients. The result of present study was consistence with result of Sun et al. [15] which found the GG genotype was present in control population. From this study, is that even if they are healthy patients, they can be protected from the CAD by confirming that they are affected with polymorphism through a PCR with RFLP test.

## Conclusion:

In conclusion, our findings show that the -IL-6-572 C/G and -IL-6-174 G/C polymorphisms are linked to the onset of hypertension. Furthermore, polymorphism was seen in control samples as well. More research, particularly with people of other races, is needed to strengthen our findings and investigate the global link between the -572 C/G and hypertension.
